# A novel compound heterozygous mutation in the DYNC2H1 gene in a Chinese family with Jeune syndrome

**DOI:** 10.1186/s41065-025-00375-x

**Published:** 2025-01-29

**Authors:** Sujie Xiong, Guangyao Hu, Yao Zhou, Fei Sun, Yanlin Ma

**Affiliations:** Key Laboratory of Reproductive Health Diseases Research and Translation of Ministry of Education & Key Laboratory of Human Reproductive Medicine and Genetic Research of Hainan Provincie & Hainan Provincial Clinical Research Center for Thalassemia, The First Affiliated Hospital of Hainan Medical University, Hainan Medical University, Haikou, Hainan 571101 China

**Keywords:** Short-rib thoracic dysplasia 3 with or without polydactyly, DYNC2H1 gene, Foetal growth restriction, Whole-exome sequencing

## Abstract

**Background:**

The dynein cytoplasmic two heavy chain 1 (DYNC2H1) gene encodes a cytoplasmic dynein subunit. Cytoplasmic dyneins transport cargo towards the minus end of microtubules and are thus termed the “retrograde” cellular motor. Mutations in DYNC2H1 are the main causative mutations of short rib-thoracic dysplasia syndrome type III with or without polydactyly (SRTD3). Early diagnosis of SRTD3 prenatally by ultrasound alone is difficult. In this case, a couple who gave birth to three consecutive babies with SRTD3 requested fertility guidance to avoid having another baby with SRTD3.

**Methods:**

Cytogenetic and molecular genetic analyses of amniotic fluid via whole-genome sequencing (WGS), routine G-banded karyotype analysis, fluorescent quantitative polymerase chain reaction, and whole-exome sequencing (WES) were performed at 19 weeks. Peripheral blood samples from the parents were also screened by Sanger sequencing for SRTD3-related mutations.

**Results:**

Two compound heterozygous mutations, c.10,594 C > T and c.7720G > A, in the DYNC2H1 gene were identified, which were inherited from the mother and father, respectively. The foetus’s mother is heterozygous for the c.10,594 C > T variant, and the foetus’s father is heterozygous for the c.7720G > A variant. The mutation c.10,594 C > T, which is a nonsense mutation believed to be pathogenic, has been previously reported. The mutation c.7720G > A, which is a missense mutation, has yet to be reported. Moreover, no chromosomal abnormalities or pathogenic copy number variations (CNVs) were detected in the foetus. The patient did not become pregnant after PGT-M and IVF-ET. This family subsequently accepted donated eggs; a successful pregnancy occurred, and a healthy girl was born.

**Conclusion:**

The compound heterogeneous mutations in DYNC2H1 ultimately accounts for the diversity of disease phenotypes reported in this study and can be used to guide future pregnancies. Our findings expand the mutation spectrum of DYNC2H1 in this rare disease and highlight the value of WES in the diagnosis of skeletal dysplasia with unclear prenatal indications.

## Introduction

Mutations in DYNC2H1 are causative mutations for short-rib thoracic dysplasia 3 with or without polydactyly (SRTD3; OMIM: 613091, also called Jeune syndrome). SRTD3 is an autosomal recessive disorder that presents radiologically with extreme narrowness of the thorax (narrow thorax), short ribs, severely shortened tubular bones with round metaphyseal ends and lateral spikes, polydactyly, increased biparietal diameter, and multiple malformations [1]. Homozygous or compound heterozygous mutations in DYNC2H1 cause substantial heterogeneity in the clinical features of SRTD3. SRTD3 can be divided into subtypes according to phenotype: short-rib polydactyly syndrome type I (SRPS1), short-rib polydactyly syndrome type II B (SRPS2B), short-rib polydactyly syndrome type III (SRPS3), asphyxiating thoracic dystrophy 3 (ATD3), polydactyly with neonatal chondrodystrophy type I, or polydactyly with neonatal chondrodystrophy type III [2].

DYNC2H1 is located at 11q22.3 and encodes dynein, cytoplasmic two, heavy chain 1. Cytoplasmic dyneins are hefty 1.5 MDa complexes composed of dimers of heavy, intermediate, light intermediate, and light chains. Cytoplasmic dyneins transport cargo towards the minus end of microtubules and are thus termed the “retrograde” cellular motor [3]. The association between cytoplasmic dyneins and ciliary intraflagellar transport (IFT), an evolutionarily conserved process, is highly significant. This protein is necessary for ciliogenesis and moving cargo towards the minus end of microtubules and positioning the Golgi complex. The protein also participates in the movement of chromosomes and the positioning of mitotic spindles during cell division and is essential for the Wnt, Notch, mTOR, and TGF-β signalling pathways [4]. Intraflagellar transport is a critical process for several mammalian organs and tissues [5].

The neonatal period is generally characterized by the lethality of SRTD3, primarily due to respiratory insufficiency resulting from the severely restricted thoracic cage. In contrast, other subtypes of SRTD are compatible with sustaining life. Prenatal diagnosis of SRTD3 is initially achieved with a two-dimensional ultrasound examination during the 18–23-week scan, at which foetal biometry and anatomy, including the skeleton and other systems such as the head and thorax, are examined in detail [6]. In the last few years, genetic testing has been increasingly used to establish a precise prenatal diagnosis [7]. Genetic diagnosis is vital in the clinic because of genetic and clinical heterogeneity. The identification of mutations in the DYNC2H1 gene would help clinicians determine the type of SRTD3 and the pathogenesis of this rare disease. In addition, clinicians could provide better guidance for subsequent pregnancies.

In the present study, we report a Chinese family with four consecutive pregnancies with a diagnosis of skeletal dysplasia on the basis of clinical and ultrasound findings. We performed whole-exome sequencing to detect potential variants and identified compound heterozygous mutations, including one known variant and one novel variant of uncertain significance, in DYNC2H1. Additionally, PGT-M technology and prenatal diagnosis were used to provide guidance to the family. The couple was not able to become pregnant successfully. Considering the advanced maternal age and abnormal delivery, this family accepted donated eggs and achieved a successful pregnancy, giving birth to a healthy girl.

## Materials and methods

### Ethical approval

Informed consent was obtained from the parents for the use of the clinical information and sample collection involved in this study. The study received approval from the Ethics Committee of the First Affiliated Hospital of Hainan Medical University. Peripheral blood samples were collected from each study participant and stored in K3EDTA tubes.

### Patients

**Fig. 1:**
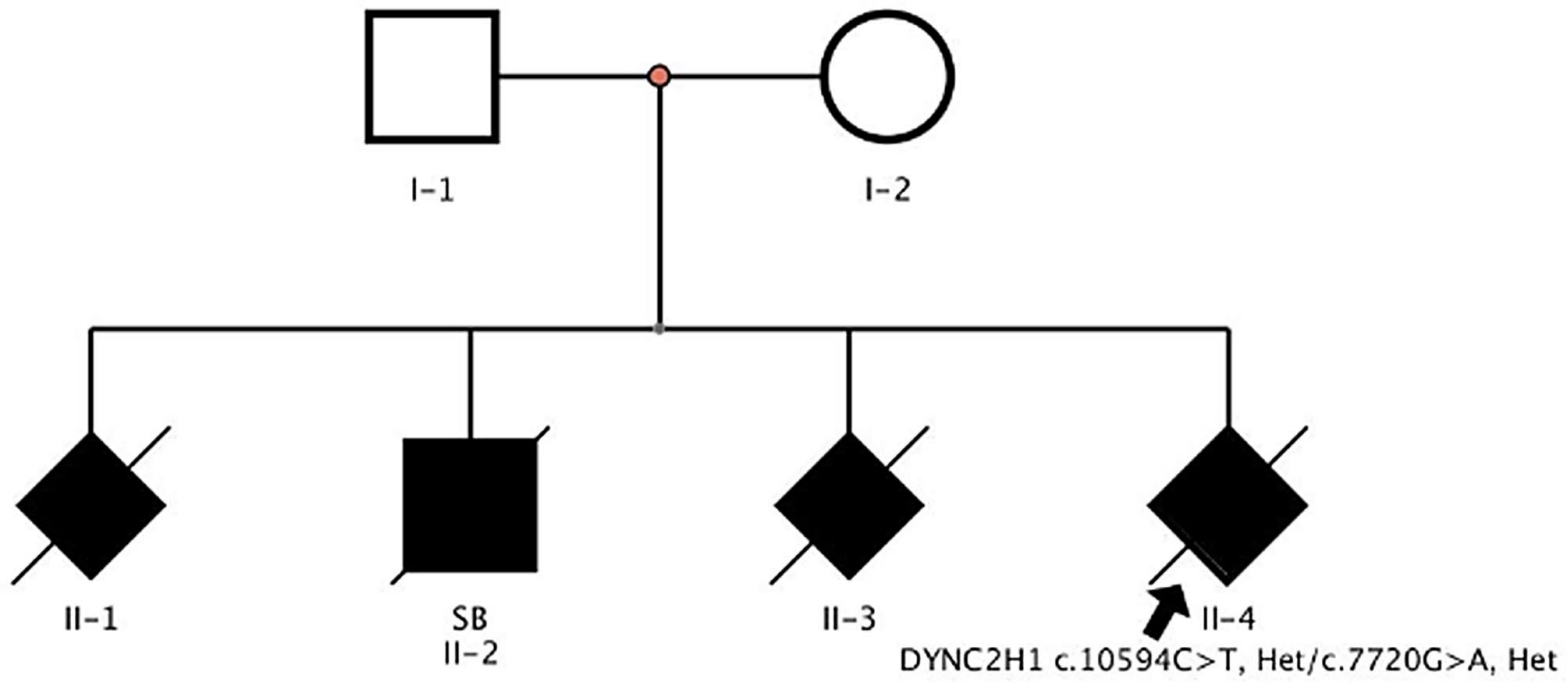
Pedigree analysis of this Chinese family

**Fig. 2:**
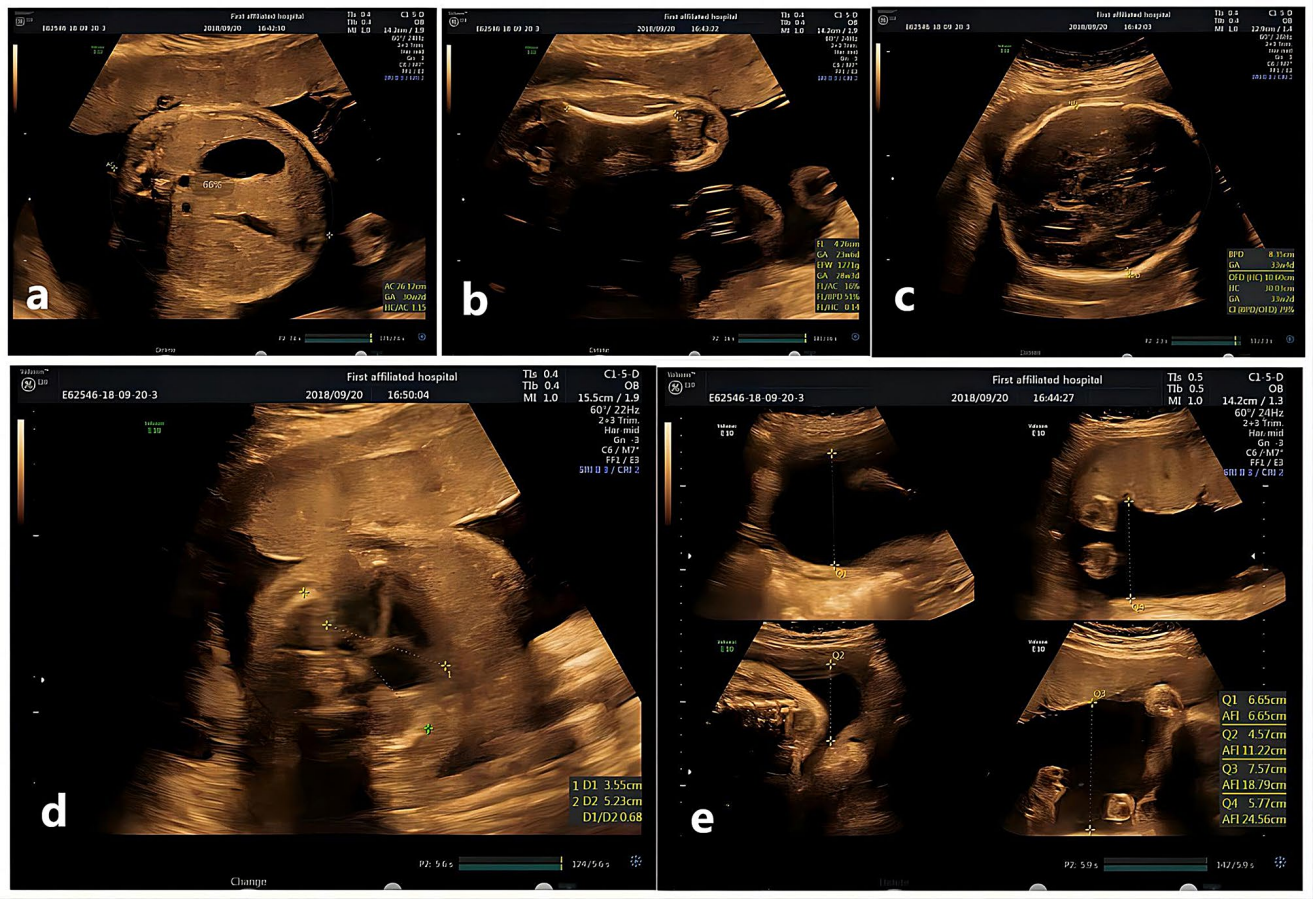
Sonographic features of the fourth fetus with DYNC2H1 compound heterozygous variants. (**a**) The abdominal circumference (AC) was equal to the length of 30 + weeks of gestation (26.12 cm). (**b**) The femur length (FL) was equal to 24 weeks of gestation (43 mm). (**c**) The head circumference (HC) was equal to the length of 33 + weeks of gestation (30 cm). (**d**) Thoracocardiac ratio:0.68 and thoracic stenosis. (**e**) Polyhydramnios (depth 25.6 cm)

The pregnant mother had a history of three abnormal pregnancies of natural conception before early 2018 (Fig. 1). All routine sonographic examinations revealed “short limb deformities and multiple anomalies of other organs” in the second and third trimesters of gestation in 2008, 2010, and 2012 (Table 1). Amniocentesis was performed in the second trimester of 2010 and 2012, revealing no abnormalities in chromosome structure or number at the 320-400 banding stage. The woman came to our hospital with her fourth pregnancy at 39 years of age. This Chinese couple was healthy and nonconsanguineous. All gestational periods included regular prenatal examinations, and Down’s screening revealed low risk. Given the previous history of abnormal pregnancy, the pregnant mother was referred to our clinic at 19 weeks of gestation for genetic counselling and subsequently underwent amniocentesis. The routine sonographic examination detected anomalies such as short limbs and a narrow thorax and did not exclude limb curvature. The couple decided to continue the pregnancy. However, a subsequent two-dimensional ultrasound examination at 30 weeks revealed that the abdominal circumference (AC) was equal to the reference circumference at 30+ weeks of gestation (Fig. 2a), the femur length (FL) was equal to the reference length at 24 weeks of gestation (Fig. 2b), and the head circumference (HC) was equal to the reference circumference at 33+ weeks of gestation (Fig. 2c). The enlarged thoracic-cardiac ratio (Fig. 2d), short femur, short ribs, and thoracic stenosis of the foetus indicated a lethal short limb deformity with polyhydramnios (depth, 25.6 cm) (Fig. 2e). Termination of the pregnancy was performed at 33 weeks’ gestation after informed consent was obtained from the parents and the Ethics Committee of The First Affiliated Hospital of Hainan Medical University.

**Table 1:** Comparisons of fetal measurement parameters between the two pregnancies at about 25 weeks of gestation in 2010 and 2018

/ (mm)	Mean: 25w	2010Y: 24^+ 6^w	2018Y: 25^+ 3^w
BPD	61.7	58(23 + w)	67(27w)
HC	228.3	211(23 + w)	244(27w)
AC	204.9	190(23 + w)	209(26 + w)
FL	44.2	30(19w)	36(21 + w)
HL	-	25(18w)	30(20w)
EFW(g)	740	468 ± 68	625 ± 91
AFD	-	58	47

**Fig. 3:**
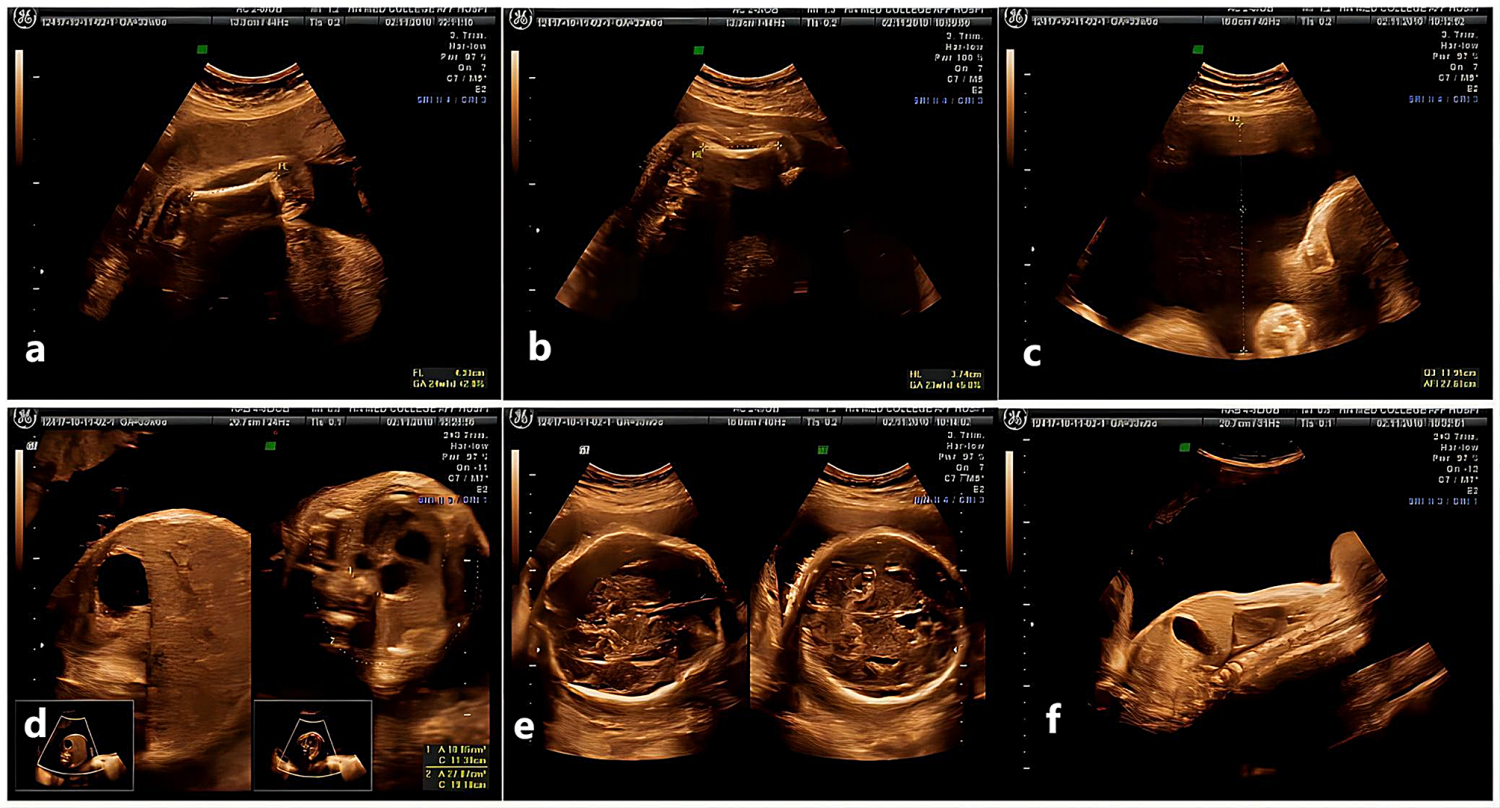
Sonographic features of the second fetus. (**a**) The FL was equal to 24 weeks of gestation (43 mm). (**b**) The HL was equal to 23 weeks of gestation (37 mm). (**c**) Polyhydramnios (depth 12.0 cm and amniotic fluid index (AFI) 44.4 cm). (**d**) The AC was equal to 29 weeks of gestation (25 cm). (**e**) The HC was equal to 33 weeks (29.7 cm). (**f**) Thoracic stenosis (22 cm)

In the second terminated pregnancy, a three-dimensional ultrasound examination at 32 weeks (third trimester of gestation) in 2010 revealed that the HC was equal to the reference circumference at 33 weeks of gestation (29.7 cm) (Fig. 3e), the FL was equal to the reference length at 24 weeks of gestation (43 mm) (Fig. 3a), the HL was equal to the reference length at 23 weeks of gestation (37 mm) (Fig. 3b), and the AC was equal to the reference circumference at 29 weeks of gestation (25 cm) (Fig. 3d). Foetal thoracostenosis (22 cm) (Fig. 3f) with a short femur and humerus (average, 15.5 cm) was considered to indicate chondrodysplasia with polyhydramnios (depth, 12.0 cm; amniotic fluid index [AFI], 44.4 cm) (Fig. 3c). The couple chose to terminate at 33 weeks of gestation. An autopsy revealed that the foetus weighed 1300 g, and the crown-heel length was approximately 47 cm with dysmorphic features, including a narrow thorax (chest circumference, 22 cm), short femur (4.3 cm), and short humerus (3.7 cm). The HC was approximately 31 cm. The positions of the heart, lungs, and thymus were normal. The heart and the great vessels did not present any abnormalities. The bilateral lungs were atelectatic. The abdominal organs were in a normal position, the liver was soft due to autolysis, the peritoneum was incomplete, and the surface of the liver was partially rough. The pelvic organs had no abnormalities in appearance. The appearance of the cranial cavity was normal, the fontanelle was not closed, and a haematoma was observed under the scalp. The umbilical blood flow was normal, and blood test results were as follows: FT3, 1.54 pmol/L; TT3, 0.67 nmol/L; and AFP, > 3340 ng/ml. X-ray imaging on 2010-11-05 revealed that the foetal bones were well ossified without abnormal bone density. The bilateral clavicles were higher than the first ribs, and the anterior ribs were slightly enlarged and bifurcated. The vertebral bodies were equally spaced in a “double-tracked” pattern with no other abnormalities.

### Basic prenatal diagnosis

To exclude the effects of maternal factors, after informed written consent was obtained for participation, DNA was extracted and purified from amniotic fluid samples from the fourth foetus for subsequent family studies and then amplified by PCR [8]. The results were analysed via capillary electrophoresis using an ABI sequencer. The samples were subsequently cultured, harvested, titrated, baked, and processed for routine G-banding, and the number and structure of chromosomes were analysed according to the ISCN2009 standards for karyotype analysis.

### Copy number variants (CNVs)

DNA was extracted from the amniotic fluid at 19 weeks of gestation after informed written consent was obtained to exclude the effects of maternal factors. High-throughput sequencing technology, combined with short tandem repeat (STR) analysis and methylation-specific PCR (MS-PCR) (BGI, Shenzhen, China), was used to detect 23 pairs of chromosomes and any aneuploidies, triploids, deletions, or duplications of more than 100 kb, five kinds of monoparental dizygotes, and intrauterine pathogen infection.

### Whole-exome sequencing (WES)

Peripheral blood was extracted from the fourth foetus and its parents after informed written consent was obtained for participation. Genomic DNA was then extracted from leukocytes following the manufacturer’s protocol. WES was conducted by a commercial next-generation sequencing (NGS) and molecular diagnostics laboratory. The exome-captured libraries were sequenced using the MGISEQ-2000 platform (BGI, Shenzhen, China) with paired-end sequencing according to the manufacturer’s instructions. A total of 3,583 genes with transparent pathogenic relationships in the OMIM database were detected and analysed via chip-captured high-throughput sequencing [9].

### Sanger sequencing

Sanger sequencing was conducted to validate the variants in the DYNC2H1 gene and to identify variants in the fourth foetus and its parents. With the fourth foetus’s genomic DNA as a template, we performed polymerase chain reaction (PCR) to amplify parts of the DYNC2H1 gene. We employed primers with the following sequences: c.10,594 C > T forward primer 5′-CTCCCCCTG GCTGAGAGT-3′ and reverse primer: 5′-TGGAACATAATTCTATGAAA GTTTGC-3′; c.7720G > A forward primer 5′-CTTTGCCCTTCT GGAGATGA-3′ and reverse primer 5′-GTGGTTTTCCATGTGGAGGT-3′. The primer sequences were designed using Primer3.0 software.

## Results

The foetal karyotype of the fourth pregnancy at 19 + weeks of gestation was normal (46, XN) according to an analysis of 20 cells, and the 100 K results revealed that a fragment of the long arm of chromosome 12 approximately 185.17 Kb long was missing in one copy, a known disease-causing mutation. QF-PCR results revealed that no apparent aneuploid variations were detected on chromosomes 13, 18, 21, X, and Y. WES results revealed the known pathogenic c.10,594 C > T mutation in the DYNC2H1 gene (NM_001080463.1) and two variants of uncertain significance (VUS), c.7720G > A and c.2154 A > G, in the DYNC2H1 gene (Table 2). First, the c.10,594 C > T variant found in exon 70 of the DYNC2H1 gene is a nonsense mutation that causes an early termination codon (p.Arg3532Term), resulting in truncated proteins, and may have a more significant impact on protein structure and function [10]. Second, the c.7720G > A variant found in exon 48 of the DYNC2H1 gene is a missense variant that substitutes valine for isoleucine at amino acid position 2574 (p.Val2574Ile). This variant is predicted to be benign by the SIFT algorithm but likely pathogenic by conservative and evolutionary predictions. Third, the c.2145 A > G variant found in exon 15 of the DYNC2H1 gene is a synonymous variation. These three mutations occur at a low frequency in the population [11].

**Table 2:** The results of WES about the fourth malformed fetus

Gene	Reference sequences	Nucleotide changes/ Mutation sites	Amino acid variations	Gene subregions	Heterozygosity	Chromosomal position	Mutation types
DYNC2H1	NM_001080463.1	c.10,594 C> T	p.Arg3532*	EX70	Het	chr11: 103,128,448	Pathogenic
DYNC2H1	NM_001080463.1	c.7720G> A	p.Val2574Ile	EX48	Het	chr11: 103,068,673	VUS
DYNC2H1	NM_001080463.1	c.2145 A> G	p.Gln715	EX15	Het	chr11: 10,300,508	VUS

**Fig. 4:**
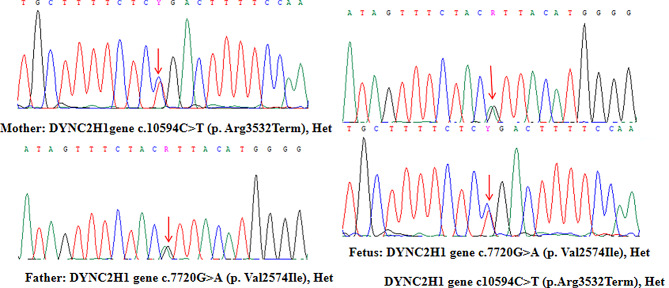
Mother: DYNC2H1 gene c.10,594 C > T (p. Arg3532Term), het. Father: DYNC2H1 gene c.7720G > A (p. Val2574Ile), het. Proband: DYNC2H1 gene c.10,594 C > T (p. Arg3532Term), het/DYNC2H1 gene c.7720G > A (p. Val2574Ile), het

WES was used to analyse samples from the fourth foetus after labour induction. The peripheral blood samples of the parents were also subjected to single-gene genetic sequencing. The genotypes and phenotypes of the parents and the foetus were compared, and two compound heterozygous mutations in the DYNC2H1 gene, c.10,594 C > T and c.7720G > A, which are inherited from the mother and father, respectively, were identified [12]. The foetus’s mother is heterozygous for the c.10,594 C > T variant, and the foetus’s father is heterozygous for the c.7720G > A variant (Fig. 4). The compound heterozygous mutations in the proband verified that the family exhibited autosomal recessive inheritance. The c.10,594 C > T mutation has been reported previously, whereas c.7720G > A is a novel mutation and was assessed according to ACMG guidelines. This mutation is located in a mutation hot spot, in which a high frequency of known pathogenic mutations are present in multiple adjacent residues. The known pathogenic variation has also been detected via trans-structure sequencing, and no corresponding mutations were detected in the control population in the 1000 Genomes Project, Exome Aggregation Consortium (EXAC), etc. The c.7720G > A variant found in exon 48 of the DYNC2H1 gene is a missense variant that substitutes valine for isoleucine at amino acid position 2574 (p.Val2574Ile). This variant is predicted to be likely pathogenic. The synonymous variation is not discussed in this article because the frequency of this locus in the population is low.

## Discussion

SRTD3 is a rare heterogeneous group of disorders characterized by thoracic dysplasia and skeletal malformations [13]. In this study, the pregnant mother had a history of three abnormal natural pregnancies, with all routine sonographic examinations in the second trimester of gestation detecting “short limb deformities and multiple anomalies of other organs”. Samples from the fourth foetus were analysed by whole-exome sequencing (WES), and two compound heterozygous variants, c.10,594 C > T and c.7720G > A, in the DYNC2H1 gene, which were inherited from the mother and father, respectively, were identified. Given the normal phenotypes of the mother and father, the mutations in this family are highly consistent with autosomal recessive monogenic inheritance.

There are two main models of skeletal development in humans: endochondral ossification, the predominant mode of bone formation in our bodies, and intramembranous ossification, which is responsible for the formation of bones such as the clavicle and skull. The DYNC2H1 gene encodes a cytoplasmic dynein subunit, a component of intraciliary transporter protein complex A [14]. During retrotranslocation, intraciliary transporter protein complex A binds to intracellular signalling molecules, and the cytoplasmic dynein subunit binds to the double microtubules at the top of the cilium, moving along the double microtubules from the top of the cilium to the bottom and participating in the recycling of ciliary proteins [15]. The anabolic bone formation process is disrupted when DYNC2H1 protein function is compromised. The clinical manifestations of this phenomenon primarily manifest as shortened ribs and limbs. These skeletal elements are mainly formed through endochondral ossification, which is dependent on the proliferation and differentiation of chondrocytes within the cartilage template through the signalling pathway, ultimately leading to the observed clinical manifestations [16].

Previous reports revealed that DYNC2H1 is the most common gene associated with SRTD3 [17, 18] and contains numerous mutation sites, one of which is the c.7720G > A variant. Missense and nonsense mutations may appear simultaneously, causing SRTD3 [19]. The mutant proteins exhibited high conservation across different species, and our preliminary study confirmed that the c.7720G > A variant is a de novo mutation. This result expanded the genetic profile of the DYNC2H1 gene in the Chinese population, especially exon 48 of this gene. Genetic diagnosis has two main applications: first, to elucidate the structure of the causative mutation in abnormalities diagnosed prenatally, and second, to identify underlying genetic defects in diseases for which there is currently no effective treatment. These applications are critical, as they can reduce the degree of psychological distress experienced by pregnant women and their partners. WES and targeted NGS are accurate and effective genetic diagnostic methods [20, 21].

The development of whole-exome sequencing technology has enabled the determination of the pathogeneses of ciliopathies [22, 23]. For SRTD3, it is challenging to determine the type of disease simply by prenatal ultrasound, and genetic diagnosis is needed. The application of genetic diagnosis technologies, such as second-generation sequencing and whole-exome sequencing, has greatly facilitated the molecular diagnosis of skeletal developmental disorders, enriched and improved the gene pool, and facilitated medical staff’s understanding of the disease; these changes are conducive to guiding clinical work and have shown that clinical diagnosis and treatment are complementary to genetic diagnosis and scientific research. As of September 2023, skeletal development disorders accounted for 7% of the PGT work at our centre. A precise genetic diagnosis can provide a theoretical basis for genetic counselling for the next generation. When a similar adverse maternal history is reported, genetic diagnosis should be carried out promptly, fertility counselling should be given, and assisted reproduction should be used in due course.

## Data Availability

No datasets were generated or analysed during the current study.

## Abbreviations

DYNC2H1: The dynein cytoplasmic two heavy chains 1
SRTD3: Short Rib-Thoracic Dysplasia Syndrome Type III with or without polydactyly
WGS: Whole gene sequencing
WES: Whole exome sequencing
CNVs: Copy number variations
SRPS1: Short-rib polydactyly syndrome type I
SRPS2B: Short-rib polydactyly syndrome type IIB
SRPS3: Short-rib polydactyly syndrome type III
ATD3: Asphyxiating thoracic dystrophy 3
IFT: Intraflagellar transport
LMP: The (e)(d)last menstrual period
EDD: The expected delivery date
hCG: Human chorionic gonadotropin
HC: Head circumference
AC: Abdominal circumference
FL: Femur length
AFI: Amniotic fluid index
QF-PCR: Quantitative fluorescence polymerase chain reaction
DNA: Deoxyribonucleic acid
VUS: Variants of uncertain significance
ACMG: American Society for Medical Genetics and Genomics
EXAC: Exome Aggregation Consortium
